# Supplementary material for: A comprehensive evaluation of long read error correction methods
Source: BMC Genomics. 2020 Dec 21;21(Suppl 6):889. doi: 10.1186/s12864-020-07227-0 (PMC7751105; doi:10.1186/s12864-020-07227-0)
Supplement: Supplementary file 1 — Additional file 1 Settings of the error correction tools and correction results on subsamples of yeast PacBio and ONT sequencing data. [file 12864_2020_7227_MOESM1_ESM.pdf]

A comprehensive evaluation of long read error correction methods: Supplementary material

Haowen Zhang[1], Chirag Jain[1], and Srinivas Aluru[1,2]

[1]School of Computational Science and Engineering, Georgia Institute of Technology, Atlanta, GA 30332, USA

[2]Institute for Data Engineering and Science, Georgia Institute of Technology, Atlanta, GA 30332, USA

Versions and configurations

The version and configuration for each error correction tool are as follows.

- Hercules: version 0.1 was used. Bowtie2 [1] was used to align compressed short reads to compressed long reads, which is suggested in the manual. The default parameters were used.
- HG-CoLoR: commit cc18a95 was used. The maximum K-mer size (−maxK) of the variable-order de Bruijn graph was set to 100 as suggested in its paper. We set top alignments reported by BLASR [2] (−bestn) to 30, 40 and 50 for *E. coli*, yeast and fruit fly data sets respectively, according to the short reads sequencing depth, which is recommended in its manual.
- FMLRC: version 0.1.2 was used. Before running FMLRC, a BWT of short reads was constructed with msBWT [3] version 0.3.0 using default parameters according to its manual. FMLRC was run with default short and long k-mer length, which is 21 and 59 respectively.
- HALC: version 1.1 was used. Prior to running HALC, AbySS [4] version 2.0.2 was used to generate contigs from short reads using 64 as k-mer length as what is suggested in its paper. HALC was run with -o to use ordinary mode so that LoRDEC is used to refine the repeat corrected regions.
- CoLoRMap: OEA mode was not tested in the experiment since it did not improve correction quality significantly but increased its runtime by about two-fold. No other parameters are available for tuning.
- Jabba: version 1.0.0 was used. Prior to running Jabba, karect [5] and btownie (`https://github.com/biointec/brownie`) were run to construct and correct de Bruijn graph with short reads. The de Bruijn k-mer size was set to 75 (-k 75) according to the user-manuals of btownie and Jabba.
- Nanocorr: the long reads were partitioned using the script provided 'partition.py'. The number of reads per file was set to 100, and the number of partition files per directory was also set to 100. The read files in the same directory were processed in parallel. To reduce disk usage, the temporary files were removed immediately after the extraction of corrected reads.
- prooveread: version 2.14.1 was used. The coverage of short reads was set according to the data sets used.
- LoRDEC: version 0.8 was built with GATB version 1.41 (`https://github.com/GATB/gatb-core`). Default parameters were used.
- ECTools: the long reads were partitioned and processed in the same way as Nanocorr. AbySS version 2.0.2 was also used to generate contigs from short reads using the same parameters that were used for HALC. Nucmer version 3.1 was used for alignment.
- LSC: version 2.0.1 was used. The default parameters were used.
- FLAS: commit 053c19b was used. The default parameters were used.
- LoRMA: version 0.5 was built with GATB version 1.4.0. LoRDEC version 0.8 was used. Default parameters were used for number of friends (-friends 7) and k-mer size (-k 19).
- Canu: version 1.7 was used. -correct was used to generate corrected reads rather than running the whole assembly pipeline. The genome size was set to the size of the reference genome. Parameter 'useGrid' was set to false to run Canu on one node. Parameter 'stopOnReadQuality' was also set to false to keep Canu running even if read quality is low. Parameters '-pacbio-raw' and '-nanopore-raw' were used for PacBio and ONT reads respectively.

Table S1: Subsamples of D2-P and D2-O

Data set	Sequencing depth	N50 (bp)	Number of reads
D2-P-10x	10x	8635	20113
D2-P-20x	20x	8665	39989
D2-P-30x	30x	8674	59928
D2-P-60x	60x	8646	119717
D2-P-90x	90x	8649	179488
D2-O-10x	10x	6976	20449
D2-O-20x	20x	6983	40720
D2-O-30x	30x	6994	61292

Table S2: Experimental results for D2-P-10x

Method	# Reads	# Bases (Mbp)	# Aligned reads	# Aligned bases (Mbp)	Maximum length (bp)	N50 (bp)	Genome fraction (%)	Alignment identity (%)	CPU time (hh:mm:ss)	Wall time (hh:mm:ss)	Memory Usage (GB)
Original	20 113	122.6	19 801	111.9	28 392	8635	99.953	87.2209	-	-	-
Non-hybrid methods											
FLAS	15 474	79.8	15 471	78.5	26 236	6363	90.204	97.4409	00:14:53	00:02:39	1.1
LoRMA	9641	11.0	9626	10.9	9370	1260	3.775	99.3906	02:11:54	00:08:51	63.7
Canu	18 747	113.4	18 614	105.2	27 582	8258	99.919	95.6321	01:43:19	00:07:16	1.7
Short-read-assembly-based methods											
HG-CoLoR	19 945	114.6	19 933	109.8	30 409	8108	99.971	99.3121	41:07:30	02:00:39	30.8
FMLRC	20 056	115.8	19 901	110.0	27 851	8149	99.972	99.3699	00:28:13	00:12:16	5.5
HALC	20 061	117.0	20 000	108.2	28 792	8231	99.971	99.0492	06:04:58	00:22:16	3.9
Jabba	16 994	90.7	16 987	90.7	28 392	7831	94.619	99.9825	00:27:21	00:03:49	21.4
LoRDEC	20 063	117.8	19 936	109.0	27 963	8296	99.968	97.9255	01:59:16	00:07:25	1.6
ECTools	11 029	79.6	11 025	79.3	27 593	8390	99.103	99.7681	56:00:03	03:31:58	4.3
Short-read-alignment-based methods											
Hercules	-	-	-	-	-	-	-	-	-	>72:00:00	-
CoLoRMap	20 110	120.2	19 984	112.3	28 299	8331	99.973	97.7314	09:05:14	00:35:53	17.6
Nanocorr	19 462	106.8	19 455	104.4	28 515	7885	99.944	98.8748	375:05:59	15:54:35	23.3
proovread	20 053	117.4	19 935	109.4	28 257	8270	99.965	98.2908	13:50:11	01:21:28	14.6
LSC	19 310	110.0	19 284	104.6	27 642	8099	99.953	96.2482	415:34:35	19:22:04	82.6

Table S3: Experimental results for D2-P-20x

Method	# Reads	# Bases (Mbp)	# Aligned reads	# Aligned bases (Mbp)	Maximum length (bp)	N50 (bp)	Genome fraction (%)	Alignment identity (%)	CPU time (hh:mm:ss)	Wall time (hh:mm:ss)	Memory Usage (GB)
Original	39 989	244.1	39 358	223.0	31 859	8665	99.976	87.2611	-	-	-
Non-hybrid methods											
FLAS	30 822	179.5	30 807	177.2	27 839	7383	99.753	98.6296	00:40:04	00:06:01	2.2
LoRMA	18 827	19.4	18 798	19.3	10 847	1095	5.388	99.2904	07:31:50	00:24:43	63.9
Canu	37 712	227.6	37 404	211.0	31 058	8279	99.974	97.0266	04:11:45	00:14:34	4.7
Short-read-assembly-based methods											
HG-CoLoR	-	-	-	-	-	-	-	-	-	-	-
FMLRC	39 879	230.6	39 574	219.3	30 884	8192	99.976	99.3929	01:12:06	00:14:38	5.5
HALC	39 893	233.0	39 777	222.1	30 871	8283	99.976	98.9419	09:43:42	01:16:27	25.5
Jabba	33 709	181.2	33 694	181.1	30 110	7934	94.973	99.9843	00:27:23	00:03:46	21.4
LoRDEC	39 895	234.6	39 630	217.0	30 871	8336	99.976	97.9517	04:19:37	00:13:12	1.6
ECTools	21 778	158.5	21 771	157.9	27 593	8457	99.483	99.7685	115:09:51	06:48:31	4.3
Short-read-alignment-based methods											
Hercules	-	-	-	-	-	-	-	-	-	>72:00:00	-
CoLoRMap	39 979	239.3	39 696	222.8	30 860	8398	99.975	97.5788	08:54:06	00:48:47	20.6
Nanocorr	37 081	204.0	37 065	199.3	28 515	7928	99.974	98.8683	748:27:25	28:25:25	23.9
proovread	39 894	234.0	39 636	217.5	30 818	8319	99.977	98.1525	21:39:23	02:31:41	47.9
LSC	28 386	162.9	28 361	155.7	28 097	8185	99.975	96.4823	900:15:49	37:05:17	101.9

Note: HG-CoLoR reported an error when correcting this dataset.

Table S4: Experimental results for D2-P-30x

Method	# Reads	# Bases (Mbp)	# Aligned reads	# Aligned bases (Mbp)	Maximum length (bp)	N50 (bp)	Genome fraction (%)	Alignment identity (%)	CPU time (hh:mm:ss)	Wall time (hh:mm:ss)	Memory Usage (GB)
Original	59 928	366.1	58 969	334.1	31 859	8674	99.976	87.2517	-	-	-
Non-hybrid methods											
FLAS	46 430	281.2	46 407	277.5	30 823	7779	99.972	99.0321	01:20:05	00:10:32	2.5
LoRMA	36 267	34.5	36 238	34.3	9973	976	9.614	99.3186	15:39:16	00:44:10	64.2
Canu	48 936	283.1	48 906	279.5	28 146	7985	99.974	99.1508	06:44:09	00:22:21	6.3
Short-read-assembly-based methods											
HG-CoLoR	59 071	340.5	59 031	325.9	45 830	8129	99.975	99.0657	109:40:58	05:00:33	52.5
FMLRC	59 762	345.7	59 288	328.6	30 884	8192	99.976	99.3896	02:05:00	00:15:03	5.5
HALC	59 784	349.3	59 598	322.8	30 864	8285	99.976	99.0676	13:38:33	02:17:41	21.7
Jabba	50 651	271.9	50 628	271.8	30 110	7897	95.104	99.9834	00:30:24	00:03:57	21.4
LoRDEC	59 795	351.7	59 392	325.2	30 871	8335	99.976	97.9507	06:29:45	00:18:34	1.6
ECTools	32 701	237.5	32 689	236.5	28 107	8437	99.598	99.7714	178:10:23	10:50:32	4.5
Short-read-alignment-based methods											
Hercules	-	-	-	-	-	-	-	-	-	>72:00:00	-
CoLoRMap	59 911	358.2	59 421	332.4	31 129	8397	99.976	97.1147	09:15:34	01:00:21	22.0
Nanocorr	56 946	313.2	56 922	306.1	28 515	7901	99.974	98.8705	1119:49:00	42:22:05	24.4
proovread	59 794	351.3	59 364	325.8	30 820	8327	99.976	97.9828	31:41:40	03:51:31	19.5
LSC	57 501	328.8	57 430	312.3	31 242	8156	99.976	96.2465	1503:43:21	56:50:09	126.9

Table S5: Experimental results for D2-P-60x

Method	# Reads	# Bases (Mbp)	# Aligned reads	# Aligned bases (Mbp)	Maximum length (bp)	N50 (bp)	Genome fraction (%)	Alignment identity (%)	CPU time (hh:mm:ss)	Wall time (hh:mm:ss)	Memory Usage (GB)
Original	119 717	731.0	117 797	666.0	33 569	8646	99.976	87.2680	-	-	-
Non-hybrid methods											
FLAS	90 214	560.0	90 154	552.3	30 926	8019	99.975	99.4142	4:08:56	0:28:08	4.6
LoRMA	327 985	413.8	327 922	413.5	14 691	1495	82.967	99.7697	53:25:06	02:21:48	66.6
Canu	62 265	322.5	62 236	319.4	27 767	7280	99.940	99.2633	05:37:49	00:19:42	6.3
Short-read-assembly-based methods											
HG-CoLoR	-	-	-	-	-	-	-	-	-	-	-
FMLRC	119 359	690.1	118 424	655.5	33 643	8178	99.976	99.3838	03:50:30	00:19:47	5.4
HALC	119 393	697.4	119 041	643.7	33 391	8266	99.976	99.0768	27:42:21	04:31:29	22.3
Jabba	101 451	544.2	101 405	543.9	30 110	7865	95.380	99.9832	00:38:22	00:04:21	21.4
LoRDEC	119 425	702.2	118 623	648.5	33 414	8318	99.976	97.9590	12:55:31	00:31:52	1.7
ECTools	65 492	473.9	65 474	472.0	28 186	8414	99.705	99.7703	386:47:01	22:13:17	4.4
Short-read-alignment-based methods											
Hercules	119 647	724.3	117 814	660.0	33 533	8575	99.976	90.1448	364:22:49	13:13:01	247.7
CoLoRMap	119 668	714.6	118 558	660.5	33 398	8401	99.976	96.5449	11:52:58	01:38:33	25.4
Nanocorr	-	-	-	-	-	-	-	-	-	>72:00:00	-
proovread	119 457	702.9	118 424	649.1	33 341	8333	99.976	97.4479	71:11:36	08:18:46	29.9
LSC	-	-	-	-	-	-	-	-	-	>72:00:00	-

Note: HG-CoLoR reported an error when correcting this dataset.

Table S6: Experimental results for D2-P-90x

Method	# Reads	# Bases (Mbp)	# Aligned reads	# Aligned bases (Mbp)	Maximum length (bp)	N50 (bp)	Genome fraction (%)	Alignment identity (%)	CPU time (hh:mm:ss)	Wall time (hh:mm:ss)	Memory Usage (GB)
Original	179 488	1095.9	176 591	998.2	35 196	8649	99.976	87.2655	-	-	-
Non-hybrid methods											
FLAS	132 435	829.5	132 330	818.4	30 200	8076	99.976	99.5446	07:52:02	00:50:35	6.3
LoRMA	473 099	848.4	472 994	848.0	19 426	2405	99.589	99.8003	112:39:38	04:42:55	69.3
Canu	64 459	459.1	64 440	454.3	28 521	8190	99.976	99.4384	09:13:31	00:30:03	6.7
Short-read-assembly-based methods											
HG-CoLoR	-	-	-	-	-	-	-	-	-	-	-
FMLRC	178 954	1034.5	177 577	982.3	33 658	8180	99.976	99.3872	05:54:38	00:24:10	5.5
HALC	179 016	1045.5	178 487	964.7	34 783	8262	99.976	99.0738	39:00:28	06:40:52	27.3
Jabba	152 100	814.6	152 021	814.2	30 141	7849	95.514	99.9831	00:34:37	00:04:36	21.4
LoRDEC	179 063	1052.7	177 874	971.8	34 896	8321	99.978	97.9513	19:51:52	00:46:31	1.9
ECTools	98 063	709.0	98 036	706.2	28 749	8408	99.728	99.7721	643:09:22	42:43:28	4.6
Short-read-alignment-based methods											
Hercules	179 444	1091.5	176 614	994.3	35 196	8619	99.976	88.4041	205:47:53	07:31:11	248.0
CoLoRMap	179 406	1071.1	177 772	990.0	35 107	8403	99.976	96.4924	15:29:41	02:21:01	30.1
Nanocorr	-	-	-	-	-	-	-	-	-	>72:00:00	-
proovread	179 133	1056.1	177 376	972.2	35 088	8347	96.814	96.8140	120:38:36	12:43:12	39.1
LSC	-	-	-	-	-	-	-	-	-	>72:00:00	-

Note: HG-CoLoR reported an error when correcting this dataset.

Table S7: Experimental results for subsamples of D2-O-10x

Method	# Reads	# Bases (Mbp)	# Aligned reads	# Aligned bases (Mbp)	Maximum length (bp)	N50 (bp)	Genome fraction (%)	Alignment identity (%)	CPU time (hh:mm:ss)	Wall time (hh:mm:ss)	Memory Usage (GB)
Original	20 225	121.6	18 472	108.4	55 374	6979	99.896	86.1285	-	-	-
Non-hybrid methods											
FLAS	16 528	82.5	16 476	81.8	17 924	5623	91.721	94.7627	00:27:46	00:03:12	0.3
LoRMA	10 650	14.2	10 488	13.9	9155	1653	6.391	97.8597	02:31:10	00:09:17	63.7
Canu	19 236	115.5	18 179	106.5	20 355	6899	99.631	94.6333	02:04:50	00:08:33	5.7
Short-read-assembly-based methods											
HG-CoLoR	-	-	-	-	-	-	-	-	-	-	-
FMLRC	20 219	121.3	19 010	113.3	55 374	6979	99.947	99.2462	00:47:07	00:11:15	2.2
HALC	20 218	122.0	19 451	110.0	55 377	7011	99.939	98.8731	05:57:43	00:22:14	4.0
Jabba	16 782	91.1	16 723	91.0	20 379	6726	94.506	99.9806	00:36:33	00:03:37	21.5
LoRDEC	20 227	122.5	18 836	110.1	55 375	7029	99.932	96.8841	01:42:07	00:08:02	1.7
ECTools	13 817	90.4	13 774	89.9	20 358	7053	98.658	99.7718	74:06:02	03:46:36	5.3
Short-read-alignment-based methods											
Hercules	-	-	-	-	-	-	-	-	-	>72:00:00	-
CoLoRMap	20 247	123.3	18 684	111.7	55 374	7051	99.936	97.3563	06:49:34	00:30:13	13.4
Nanocorr	17 728	103.0	17 675	102.2	20 532	6929	99.879	98.7603	631:40:01	24:23:08	14.5
proovread	20 223	121.8	18 693	109.9	55 374	6994	99.942	97.6638	11:52:55	01:11:43	14.1
LSC	17 103	99.1	17 041	98.1	20 452	6806	99.870	94.8926	676:27:14	27:08:41	147.8

Note: HG-CoLoR reported an error when correcting this dataset.

Table S8: Experimental results for subsamples of D2-O-20x

Method	# Reads	# Bases (Mbp)	# Aligned reads	# Aligned bases (Mbp)	Maximum length (bp)	N50 (bp)	Genome fraction (%)	Alignment identity (%)	CPU time (hh:mm:ss)	Wall time (hh:mm:ss)	Memory Usage (GB)
Original	40 299	242.4	36 821	215.8	55 374	6985	99.966	86.1341	-	-	-
Non-hybrid methods											
FLAS	33 868	193.7	33 763	192.3	21 785	6374	99.660	96.1600	01:19:51	00:07:54	1.9
LoRMA	52 979	51.4	52 785	50.9	9147	997	35.481	98.2431	09:07:28	00:29:41	64.2
Canu	38 609	232.3	36 309	213.8	42 911	6913	99.856	95.6186	04:59:32	00:14:45	7.2
Short-read-assembly-based methods											
HG-CoLoR	-	-	-	-	-	-	-	-	-	-	-
FMLRC	40 288	241.8	37 931	225.6	55 374	6979	99.967	99.2302	01:21:36	00:12:19	1.3
HALC	40 293	243.3	38 719	219.2	55 378	7011	99.972	98.8497	16:35:41	01:26:22	4.1
Jabba	33 467	181.4	33 333	181.1	20 379	6727	94.950	99.9825	00:39:48	00:03:46	21.5
LoRDEC	40 301	244.2	37 535	219.2	55 375	7033	99.968	96.8777	03:33:04	00:12:56	1.8
ECTools	27 436	179.6	27 354	178.7	20 358	7059	99.080	99.7703	144:19:40	06:48:58	5.6
Short-read-alignment-based methods											
Hercules	-	-	-	-	-	-	-	-	-	>72:00:00	-
CoLoRMap	40 333	245.6	37 127	221.8	55 374	7054	99.971	97.0285	07:36:44	00:45:04	20.7
Nanocorr	35 299	205.0	35 187	203.4	23 384	6929	99.965	98.7633	1260:36:46	47:38:41	15.2
proovread	40 297	242.9	37 190	218.7	55 374	6995	99.971	97.3920	20:03:49	02:15:13	15.9
LSC	34 067	197.5	33 943	195.4	23 499	6810	99.959	94.9089	1323:52:37	49:05:31	106.5

Note: HG-CoLoR reported an error when correcting this dataset.

Table S9: Experimental results for subsamples of D2-O-30x

Method	# Reads	# Bases (Mbp)	# Aligned reads	# Aligned bases (Mbp)	Maximum length (bp)	N50 (bp)	Genome fraction (%)	Alignment identity (%)	CPU time (hh:mm:ss)	Wall time (hh:mm:ss)	Memory Usage (GB)
Original	60 648	365.4	55 402	325.4	55 374	6997	99.971	86.1812	-	-	-
Non-hybrid methods											
FLAS	50 245	300.1	50 085	298.0	21 411	6686	99.909	96.5553	02:30:09	00:13:51	2.7
LoRMA	154 864	146.3	154 668	145.7	9512	972	78.164	98.4020	20:18:16	00:59:45	65.3
Canu	49 872	302.3	49 748	300.2	23 194	6966	99.880	97.2080	08:40:23	00:26:48	6.7
Short-read-assembly-based methods											
HG-CoLoR	-	-	-	-	-	-	-	-	-	-	-
FMLRC	60 635	364.5	57 134	340.2	55 374	6990	99.970	99.2438	01:56:19	00:13:31	2.2
HALC	60 644	366.8	58 310	330.6	55 377	7023	99.972	98.8575	23:56:13	01:43:55	4.1
Jabba	50 474	273.4	50 271	272.9	20 379	6732	95.124	99.9816	00:44:06	00:03:59	21.5
LoRDEC	60 652	368.1	56 507	330.5	55 375	7047	99.971	96.9152	05:18:57	00:15:32	1.9
ECTools	41 303	270.8	41 182	269.5	20 358	7073	99.174	99.7668	216:17:19	10:20:44	5.7
Short-read-alignment-based methods											
Hercules	-	-	-	-	-	-	-	-	-	>72:00:00	-
CoLoRMap	60 693	369.5	55 812	333.0	55 374	7055	99.972	96.5312	08:30:17	00:58:56	22.9
Nanocorr	53 159	309.4	52 994	306.9	23 384	6947	99.971	98.7687	1898:17:48	71:13:41	15.0
proovread	60 649	366.2	55 910	329.4	55 374	7009	99.974	97.2014	30:07:31	03:25:32	19.4
LSC	-	-	-	-	-	-	-	-	-	>72:00:00	-

Note: HG-CoLoR reported an error when correcting this dataset.

References

[1] Ben Langmead and Steven L Salzberg. Fast gapped-read alignment with bowtie 2. *Nature methods*, 9(4):357, 2012.

[2] Mark J Chaisson and Glenn Tesler. Mapping single molecule sequencing reads using basic local alignment with successive refinement (blasr): application and theory. *BMC bioinformatics*, 13(1):238, 2012.

[3] James Holt and Leonard McMillan. Merging of multi-string bwts with applications. *Bioinformatics*, 30(24):3524–3531, 2014.

[4] Shaun D Jackman, Benjamin P Vandervalk, Hamid Mohamadi, Justin Chu, Sarah Yeo, S Austin Hammond, Golnaz Jahesh, Hamza Khan, Lauren Coombe, Rene L Warren, et al. Abyss 2.0: resource-efficient assembly of large genomes using a bloom filter. *Genome research*, 27(5):768–777, 2017.

[5] Amin Allam, Panos Kalnis, and Victor Solovyev. Karect: accurate correction of substitution, insertion and deletion errors for next-generation sequencing data. *Bioinformatics*, 31(21):3421–3428, 2015.
